# Evaluation of Undergraduate Dental Students’ Opinions on the Use of Digital Versus Conventional Design in Prosthodontics

**DOI:** 10.3390/dj13060242

**Published:** 2025-05-29

**Authors:** Lucian Toma Ciocan, Mihaela Pantea, Vlad Gabriel Vasilescu, Ana Maria Cristina Țâncu, Ruxandra Sfeatcu, Andreea Cristiana Didilescu, Alexandra Ripszky, Alexandra Popa, Silviu Mirel Pițuru, Marina Imre

**Affiliations:** 1 Discipline of Dental Prostheses Technology, Faculty of Dentistry, “Carol Davila” University of Medicine and Pharmacy, 37 Dionisie Lupu Street, District 2, 020021 Bucharest, Romania; lucian.ciocan@umfcd.ro; 2 Discipline of Prosthodontics, Faculty of Dentistry, “Carol Davila” University of Medicine and Pharmacy, 37 Dionisie Lupu Street, District 2, 020021 Bucharest, Romania; anamaria.tancu@umfcd.ro (A.M.C.Ț.); marina.imre@umfcd.ro (M.I.); 3 Discipline of Oral Health and Community Dentistry, Faculty of Dentistry, “Carol Davila” University of Medicine and Pharmacy, 37 Dionisie Lupu Street, District 2, 020021 Bucharest, Romania; ruxandra.sfeatcu@umfcd.ro; 4 Discipline of Embryology and Microbiology, Faculty of Dentistry, “Carol Davila” University of Medicine and Pharmacy, 8 Eroii Sanitari Boulevard, 050474 Bucharest, Romania; andreea.didilescu@umfcd.ro; 5 Department of Biochemistry, Faculty of Dental Medicine, “Carol Davila” University of Medicine and Pharmacy, 8 Eroilor Sanitari Blvd, 050474 Bucharest, Romania; alexandra.ripszky@umfcd.ro (A.R.); alexandra.popa@drd.umfcd.ro (A.P.); 6 Discipline of Organization, Professional Legislation and Dental Office Management, Faculty of Dentistry, “Carol Davila” University of Medicine and Pharmacy, 37 Dionisie Lupu Street, District 2, 020021 Bucharest, Romania; silviu.pituru@umfcd.ro

**Keywords:** digital dentistry, CAD/CAM, dental students, dental technology, prosthodontics, conventional design, digital design, digital technologies

## Abstract

**Background/Objectives**: The integration of digital technologies into dental education is becoming increasingly important, particularly in prosthodontics, where digital design tools offer enhanced precision and efficiency. This study aimed to evaluate second-year dental students’ perceptions regarding conventional versus digital design in prosthodontics, assessing their theoretical knowledge, practical skills, and attitudes toward these approaches. **Methods**: A total of 141 dental students enrolled in Faculty of Dentistry from “Carol Davila” University of Medicine and Pharmacy, Bucharest, Romania, participated in an online survey consisting of 19 questions evaluating their understanding, preferences, attitudes, and expectations regarding digital and conventional prosthodontic workflows. Additionally, students’ practical exam grades and task completion times for both conventional and digital design methods were evaluated. **Results**: Participating students reported sufficient understanding of theoretical concepts in both conventional (92.9%) and digital design (91.5%). A significant proportion (78.7%) felt confident in their practical skills for conventional design, while 78% expressed the same for digital design. Statistically significant correlations indicated that students who believed digital design could replace conventional methods associated digital design with greater accuracy (*p* = 0.020), predictability (*p* = 0.048), and sustainability (*p* = 0.032). Students who believed they had acquired enough skills in digital design responded more frequently that the time allocation for digital design by the university was sufficient (*p* < 0.001). Moreover, students scored significantly higher in digital design practical exams compared to conventional design (*p* < 0.001). Task completion times were also shorter for digital workflows, further supporting their efficiency. **Conclusions**: The findings suggest that students are highly receptive to digital technologies in prosthodontics, favoring digital workflows over conventional techniques. These results highlight the need for continued integration of digital tools into dental curricula to enhance students’ competency and prepare them for modern clinical practice.

## 1. Introduction

The use of digital technologies in all branches of dentistry has become increasingly widespread, significantly impacting the precision, efficiency, and reproducibility of treatments. The field of prosthodontics has undergone significant transformation with the integration of digital technologies, particularly computer-aided design/computer-aided manufacturing (CAD/CAM) systems facilitating the design and fabrication of dental restorations with superior accuracy compared to conventional methods [1]. These advancements have enhanced workflow efficiency, precision, and communication among dental professionals. These innovations not only enhance treatment quality but also improve patient experience by reducing the time required for restorations and increasing the predictability of outcomes [2]. Recent studies have extensively explored the impact of digital design in prosthodontics compared to conventional methods, highlighting its advantages and the growing preference for digital approaches among students and practitioners.

A comprehensive review [1] comparing digital and conventional impression techniques highlighted the superior accuracy and efficiency of digital methods in the fixed prosthodontic treatment outcomes. Similar findings were reported by other authors, who found that both clinicians and students perceived digital impressions as superior in terms of accuracy and ease of use [2]. Another study further supported this view, indicating that digital workflows reduce treatment time and increase cost-effectiveness compared to traditional approaches [3]. Additionally, some research demonstrated that digital impression techniques provide better reproducibility and eliminate many of the errors associated with conventional methods [4,5].

From an educational perspective, integrating digital technologies into the university curriculum has been shown to enhance both student engagement and confidence in using these tools in their future clinical practice. Some authors observed that students with greater exposure to digital dentistry during university training showed a stronger inclination toward incorporating digital tools into their future practice [6]. Similarly, others assessed student opinions on digital preparation assessment and noted a preference for digital evaluation tools over traditional methods due to their objectivity and reproducibility [7]. These findings suggest that digital technologies enhance student engagement and confidence in prosthodontic procedures. One article further reinforced this perspective by discussing how three-dimensional digital technologies improve dental training and procedural accuracy and efficiency [8].

Despite these benefits, some studies have highlighted potential challenges in adopting digital prosthodontics and reported that while digital workflows streamline the design and fabrication process, they require a significant initial investment in equipment and training [9]. Others found that students needed additional time to develop proficiency in CAD software compared to traditional wax-up techniques [10]. Furthermore, concerns about the adequacy of training time for digital techniques have been raised. It was noted that students often felt that the available training time was insufficient [6], a concern echoed by others, who examined the marginal and internal fit of zirconia prostheses fabricated using both digital and conventional impressions, emphasizing the learning curve associated with digital techniques [11].

Moreover, studies explored the evolution of intraoral scanners, demonstrating how digital impressions have revolutionized clinical workflows and patient experiences [5,12]. However, they also acknowledged that adaptation to digital workflows varies among clinicians and students based on their exposure and the quality of their training. Additionally, digital and conventional impression techniques for implant-supported prostheses were compared, concluding that digital workflows provided better clinical outcomes in terms of marginal fit and patient comfort [13].

Moreover, recent literature confirms the benefits of CAD/CAM technologies and additive manufacturing methods in dental restorations. Current research highlights that these technologies offer significant advantages in the design and fabrication of removable dental prostheses, contributing to a reduction in the number of clinical visits required and improving the adaptation of restorations to individual patient characteristics [14]. Studies on the use of selective laser melting in prosthesis fabrication have demonstrated improved precision and reproducibility of prosthetic structures, providing a viable alternative to conventional techniques [15]. These findings support the progressive adoption of digital technologies in dental practice and highlight the necessity of proper student training in these innovative methods.

These advantages have led dental educators to integrate digital dental technology into the university dental curriculum. Today’s dental students, having been raised in a digital era, are proficient with new technologies. They adapt quickly and are often more comfortable with digital methods than with traditional ones.

In the context of the above mentioned aspects, the aim of this study was to assess the perceptions of dental students in the Faculty of Dentistry, Bucharest, Romania, regarding their knowledge, attitude, and interest toward conventional versus digital design in prosthodontics, using a survey questionnaire. Furthermore, we analyzed students’ academic performance in practical semester exams for both digital and conventional design methods and evaluated the time required to complete practical tasks. By comparing our findings with prior studies, this research offers insights into digital dental education and suggests ways to optimize its integration into university curricula.

## 2. Materials and Methods

### 2.1. The Survey

#### 2.1.1. Survey Methodology and Ethical Approval

This survey was approved by the Scientific Research Ethics Committee of “Carol Davila” University of Medicine and Pharmacy, Bucharest, Romania (project identification code: PO-35-F-03; protocol number: 2682; date: 2 February 2024). The scientific study was conducted in accordance with the Declaration of Helsinki of 1975, revised in 2013. The students’ opinion on the use of digital design versus conventional design in prosthetic dentistry was assessed via a questionnaire, administrated as a Google form, sent via email. Participants selected for inclusion in this study were extended invitations to complete the questionnaire and were informed about the survey in adherence to the World Medical Association Declaration of Helsinki and the current European privacy regulations. In the introduction section of the questionnaire, specific details were provided: the scientific aim of the study, the remark that the questionnaire was anonymous and that the students have their right to interrupt the completion of the form at any moment in case of withdrawal. Subjects invited to take part in this study received an email containing written details about the study and the informed consent form. Students who agreed to participate in the study expressed their consent by completing the survey. Additionally, to publish this paper, informed consent was obtained from the subjects involved in the study. No personal information was gathered via the form, and being an anonymous web survey, no sensitive data were collected. The questionnaire was safeguarded to ensure it could only be completed once by each participant.

Students were informed both verbally and in writing that their participation was optional and voluntary and would not affect their grades in any way. An independent party not involved with the study assigned each student a unique participant number derived from their consent forms. Consequently, the survey responses were kept anonymous from the principal investigators of the study.

#### 2.1.2. Selection of Participants

This study was designed to be a pilot study and was conducted among second-year dental students who were undergoing theoretical and practical training in the Department of Dental Prostheses Technology, Faculty of Dentistry, “Carol Davila” University of Medicine and Pharmacy, Bucharest, Romania. The inclusion criteria for participants were as follows: second-year undergraduate dental students; students aged 18 years or older; students who had completed both the theoretical and practical training in Dental Prostheses Technology discipline over an entire semester (14 weeks), attending the theoretical courses two hours per week and the practical training sessions four hours per week; students that had already participated in the practical semester exams related to the digital and conventional design in prosthodontics, in the same faculty department; and any gender. The exclusion criteria were students who had not completed their training in Dental Prostheses Technology for an entire semester; students who had not participated in the practical semester exams; students unwilling to participate in the study; and lack of cooperation with the responsible individuals or study investigators.

The opinions of these students regarding the use of conventional fabrication of wax patterns for fixed dental prosthetic restorations versus the use of digital technology (CAD/computer-aided design) for designing dental prosthetic restorations were assessed via an online questionnaire, as previously mentioned.

Second-year dental students at the Faculty of Dentistry in Bucharest, Romania, typically receive preclinical education in dental prostheses technology as part of their curriculum. However, beginning in December 2022, a more advanced CAD/CAM theoretical and practical training was introduced, integrated and developed into their curriculum for an entire semester, following the university’s launch of the Laboratory for Digital Technologies in Dentistry, allowing students to engage with these topics in a more comprehensive manner. The manual dedicated to the Discipline of Dental Prostheses Technology was also revised to include information and instructions on using CAD/CAM technology to scan, design, and produce prosthetic restorations. As for design in prosthodontics, the curriculum corresponding to this discipline includes, in relatively equal proportions, theoretical and practical concepts regarding both digital and conventional design in prosthodontics. Students were provided with theoretical courses (lectures) and tutorial videos created by the faculty members for specific procedures in conventional and digital design in prosthodontics; students also participated in the correspondent practical training under the supervision of faculty members who provided support during the conventional or digital design procedures. The present study focuses on second-year dental students, who were at the beginning of their clinical education and had limited experience in performing dental procedures. However, it was of particular interest to us to capture their immediate post-semester feedback, especially as this group benefited from improved conditions for CAD/CAM training.

During that semester, a total of 143 students were enrolled in the Dental Prostheses Technology theoretical course and practical training. All students were invited to participate in the survey and 141 voluntarily agreed, indicating a participation acceptance rate of 98.6%. All 141 participants completed the questionnaire in full. As previously noted, the questionnaire was secured to allow only one submission per participant.

#### 2.1.3. Survey Questionnaire

The questionnaire used for the assessment (as presented in Supplementary Material Table S1) was formed of 19 items represented by single-choice questions. The questions referred to the following main aspects:

(1) Socio-demographic details (age, gender, year of study—first 3 questions);
(2) Participants’ opinions regarding their knowledge and practical skills acquired during the semester in conventional and digital design in prosthodontics (questions 4 to 9);
(3) Students attitudes toward digital and conventional design in prosthodontics (questions 10 to 17);
(4) Participants’ motivation, focusing on their perceptions regarding the future trajectory of CAD (computer-aided design) technology in dentistry and its potential continued integration into dental university education (questions 18 and 19).

To ensure the face validity and content validity of the survey prior to its distribution, five faculty members with at least 5 years of experience teaching digital dentistry-related subjects were invited to review the survey. They were provided with a paper survey and asked to assess the relevance of the survey questions in relation to the study’s objectives using a 5-point Likert scale, ranging from “very irrelevant” to “very relevant”. Their responses were collected and tabulated, and the relevance of each question was assessed individually. Questions with an agreement of at least 80% among the five faculty members were considered valid for inclusion in the survey. Questions with less than 80% agreement underwent revision and were either re-evaluated by the faculty for inclusion or excluded from the study. Students were supposed to answer to the questions with “I agree” or “I disagree”. The estimated time needed to fill the questionnaire was about 5 min.

### 2.2. The Practical Semester Exams: Student Grades and Tasks Completion Time

We collected the grades obtained by the participants in our study in the practical exams for both conventional and digital design. The practical exams consisted in the execution of a conventional wax pattern and a digital design for a provisional fixed dental restoration on the first upper left central incisor (# 2.1). A didactic dental model (Frasaco AN3, Frasaco GmbH, Tettnang, Germany), which presented tooth # 2.1 prepared for an all-ceramic crown, with an uniform 2 mm occlusal reduction and a 1 mm shoulder finish-line, was used as the reference. This reference model was scanned and the digital dental model obtained was used for the digital design exam. Based on the digital dental model, we also obtained 3D-printed models that were used for the conventional design exam. During the practical exam for the digital design in prosthodontics, students proceeded with the digital design procedures, finally conceiving their own digital design for the provisional fixed dental restoration on the first upper left central incisor (# 2.1), on the digital didactic dental model, using Medit Link software v.3.1.0 (MeditLink, Seoul, Republic of Korea). The crowns designed by the students were exported in the standard tessellation language (STL) format to two evaluating teachers. The evaluators had at least three years of experience teaching Dental Prostheses Technology and had been calibrated based on grading criteria for that course. The projects were evaluated for occlusion (including occlusal contacts and palatal surface anatomy), proximal contacts, marginal cervical fit, internal fit, general aspect morphology, buccal surface view, lingual surface view, interproximal surface view, embrasures, and overall finish. During the practical exam for the conventional design in prosthodontics, students were asked to wax a crown for tooth # 2.1 using traditional methods, by combining additive techniques and carving in order to achieve a proper crown design. The students’ projects were evaluated in the same manner as the digital ones. Successively, grades were allocated for each student, for each practical exam. The arithmetic mean of each of these types of grades was calculated, resulting in two “median grading-scores”, which corresponded to the two practical exams: a median grading score corresponding to the practical exam for conventional design and a second median grading score corresponding to the practical exam in digital design.

Furthermore, we recorded and analyzed the time taken by participating students to complete the practical tasks during the practical semester exams. Throughout the practical exams, the time (number of minutes) spent on both the digital dental project and the conventional project by each participating student was recorded by the teaching staff. Subsequently, the arithmetic mean of these times was calculated, resulting in two “median time-scores”, which corresponded to the two types of practical exams: a median time-score corresponding to the average time designated for the digital design and a second median time-score corresponding to an average time for the conventional project.

### 2.3. Data Analysis

All the data collected from the 19 questions of the survey were analyzed using IBM SPSS Statistics 25 and illustrated using Microsoft Office Excel/Word 2021. Quantitative variables were tested for normal distribution using the Shapiro–Wilk test and were written as averages with standard deviations or medians with interquartile ranges. Qualitative variables were written as counts or percentages, and differences between groups were tested using Fisher’s exact tests. Quantitative independent variables with non-parametric distribution were tested between groups using Mann–Whitney U. Correlations between quantitative variables with non-parametric distribution were measured using Spearman’s rho correlation coefficients. We additionally analyzed and compared the grades achieved by the participants in both digital and conventional design in prosthodontics during the practical semester exams, and the time taken to complete the practical tasks, and we statistically correlated these results with those obtained from the survey.

## 3. Results

### 3.1. The Survey’s Results

All participants were in the second year of study (100%) and their mean age was 20.38 ± 1.22 years (median = 20 years). Most of the students were women, representing 71.63% of participants (101 persons); 35 students (24.82% of participants) were males, and 5 students (3.55% of participants) did not declare their gender.

**Figure 1:**
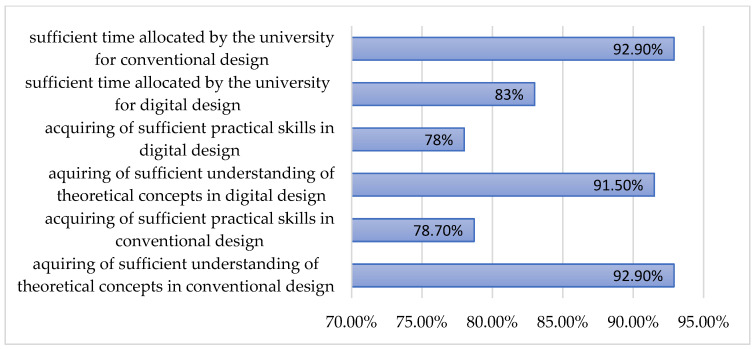
The perception of participants concerning their theoretical knowledge and practical skills in digital and conventional design in prosthodontics.

**Figure 2:**
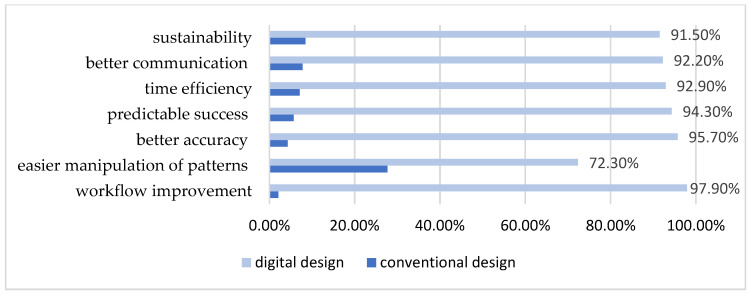
Students attitudes toward digital and conventional design in prosthodontics.

Data from Figure 1 and Figure 2 show the distribution of the students according to the answers in the survey. The responses in the survey show that most of the students considered that they acquired sufficient theoretical concepts in conventional workflow (92.9%) and in digital design (91.5%); 78.7% of the students considered that they acquired sufficient practical skills in conventional design in prosthodontics and 78% of them considered they acquired sufficient practical skills in digital design in prosthodontics. The allocated time for the practical training for conventional design in prosthodontics was sufficient for 92.9% of students, and only for 83% of them for the digital design (Figure 1).

All of the students considered that CAD is useful in medical applications (100%). Compared to conventional design, most of the students answered that digital design in prosthodontics could improve the workflow stages involved in obtaining dental prosthetic restorations (97.9%), increases the accuracy of dental prosthetic restorations (95.7%), provides a predictable success (94.3%), is time efficient (92.9%) and sustainable (91.5%), and also allows for better communication between dental professionals (92.2%) (Figure 2); however, only 72.3% of students responded that the manipulation on prosthetic patterns is easier in CAD (Figure 2). Moreover, most of the students considered that digital design could replace conventional design for dental prosthetic restorations in the future (88.7%) and would have interest in the integration of digital technology into their university education and future dental practice (96.5%).

Table 1 presents students’ responses according to their agreement with the potential replacement of conventional design by CAD (with the variable stratified into two column groups: students who disagree and those who agree) and according to their perceptions of specific advantages of digital design—ranging from workflow improvement to sustainability—each categorized as either “Present” (students who perceive the benefit) or “Absent” (students who do not). The differences observed between the groups of students were statistically significant in case of some of the analyzed items (*p* < 0.05), showing that students who considered that digital design increases the accuracy of dental prosthetic restorations (97.6% vs. 81.2%, *p* = 0.020), provides a predictable success (96% vs. 81.2%, *p* = 0.048), and optimizes resource utilization (93.6% vs. 75%, *p* = 0.032) more frequently responded that conventional design in prosthodontics could be replace by digital design in the future.

Table 2 shows the distribution of the students according to the answers regarding the advantage of easier pattern manipulation as a benefit of digital design and interest for future integration of CAD technologies into university education and dental practice. This table uses a structure with columns representing students’ interest or lack of interest in the future integration of CAD technologies, and rows indicating whether they perceive easier pattern manipulation as a benefit of digital design. The differences observed between the student’ responses were not statistically significant in most of the analyzed items (*p* > 0.05), except for one of them: students who considered that digital design, compared to conventional design, would help in easier pattern manipulation, responded more frequently that they would be interested in the future integration of digital design technology into their university education and dental practice (74.3% vs. 20%, *p* = 0.021).

Data from Table 3 shows the distribution of the students according to the answers regarding the acquirement of sufficient theoretical knowledge and practical skills in conventional and digital design in prosthodontics, and the time allocated by the university for these aspects. This table displays the relationship between students’ perceptions of whether the time allocated by the university for conventional and digital design was sufficient (columns), and whether they believe they acquired adequate theoretical or practical skills in each method (rows). The differences observed between the groups of students were not statistically significant when the associations between the answers regarding the acquirement of sufficient theoretical understanding of concepts (*p* = 0.533) or practical skills (*p* = 0.689) in conventional workflow, and the time allocation were tested. Students that considered they acquired sufficient understanding of theoretical concepts (96.6% vs. 66.7%, *p* < 0.001) or practical skills (85.5% vs. 41.7%, *p* < 0.001) in digital design had significantly more frequently responded that the time allocated by the university was sufficient in comparison to students that considered they did not acquire sufficient theoretical knowledge or practical skills in CAD.

### 3.2. Results of the Practical Semester Exams: Student Grades and Tasks Completion Time

#### 3.2.1. Analysis of the Student Grades

Data from Table 4 show the comparison between the median grading scores corresponding to the practical exam for conventional and digital design in prosthodontics. The difference between the two median grading scores was statistically significant (*p* < 0.001), showing that the grades for the digital design exam (median grading scores = 8.23) were significantly higher (median = 8, IQR = 7.75–9) in comparison to the grades obtained in the conventional exam (median grading-score = 7.61) (median = 8, IQR = 6–9), the difference being significant (mean = 0.61 ± 1.65, median = 0, IQR = −1–2).

Data from Table 5 show the comparison of the grading scores in the analyzed students according to their opinion about the skills acquirement. This table shows a non-parametric comparison of the students’ grading scores using Mann–Whitney U tests. Groups were stratified based on whether students reported acquiring, or not acquiring, theoretical or practical skills in either conventional or digital design. Differences in conventional grading scores were not significant between students who agreed or not about their sufficiency in acquired theoretical skills (*p* = 0.880) or practical skills (*p* = 0.408). However, the students who considered that they acquired sufficient understanding of theoretical concepts in CAD (median = 8, IQR = 8–9 vs. median = 7, IQR = 7–8.75, *p* = 0.028) or practical skills in CAD (median = 8, IQR = 8–9 vs. median = 7, IQR = 7–9, *p* = 0.001) had significantly higher grades in digital design in comparison to the students who considered that they did not acquire enough skills.

#### 3.2.2. Analysis of Task Completion Time

The analysis of the median time scores acquired for the execution of a conventional wax pattern and a digital design for a provisional fixed dental restoration on the first upper left central incisor (# 2.1) (“average time for digital design” and “average time for the conventional project”) highlighted that the digital designs (projects) carried out by the participating students during the practical semester exams required less time compared to conventional projects (Table 6). Students spent on average 22–41 min more on conventional projects than on digital ones.

## 4. Discussion

Given the advantages that digital dental technology brings to the field of dentistry, it is natural that that education in digital dentistry has witnessed widespread popularity on various levels, including among teaching staff and educational researchers [6,7]. In this study, our primary focus was to comparatively assess the perceptions of second-year dental students regarding their knowledge, practical skills, attitude, and interest toward conventional versus digital design in prosthodontics. Additionally, the grades obtained by the participants in the practical semester exams for both digital and conventional design in prosthodontics, along with the time required to complete the practical tasks, were also analyzed. Students participating in the study indicated a sufficient understanding of theoretical concepts in conventional (92.9%) and digital design (91.5%) in prosthodontics. We found statistically significant correlations in certain aspects of our analysis, which highlight the advantages of digital design as perceived by the participants: students who considered that digital design could replace conventional design in the future responded that digital design is more accurate (*p* = 0.020), predictable (*p* = 0.048), and sustainable (*p* = 0.032); those who believed they had acquired sufficient understanding of theoretical concepts and sufficient practical skills in digital design responded more frequently that the university allocated sufficient time for digital design (*p* < 0.001). Additionally, the grades obtained by the participating students in the digital design exam were significantly higher in comparison to the grades obtained in the conventional exam (*p* < 0.001). Furthermore, the digital projects carried out by the participating students during the practical semester exams required less time compared to conventional projects. The results obtained in our study are in line with the ones found in the scientific dental literature.

### 4.1. Digital Versus Conventional Prosthodontic Design

The comparison between digital and conventional prosthodontic design has been widely discussed in the scientific literature, with multiple studies assessing accuracy, efficiency, and user preference associated with digital workflows. Our results align with previous literature, which reported that digital impressions minimize errors and are easier to use [1,2]. These findings support our observation that digital design improves workflow precision and efficiency (*p* = 0.020). The literature highlights that digital methods reduce treatment and training time [3]. In our study, students completed digital projects significantly faster than conventional ones, a result echoed in their improved grades. Although mastering CAD software may initially require more effort, the learning curve is offset by long-term gains in efficiency [10]. Students’ interest in integrating digital tools into future practice (96.5%) also reflects broader trends [6]. However, our study did not find a significant correlation between theoretical knowledge and interest in CAD adoption, diverging from Sheba’s findings. Training time and cost remain key concerns. Some authors stress the importance of structured training and institutional investment [9,11]. Our results support this, as students who felt confident in digital design were more likely to consider their training time sufficient (*p* < 0.001). In summary, our data reinforce the value of digital integration in dental education, while also acknowledging the need for careful planning, training, and curriculum support.

### 4.2. Replacement of Conventional Design with CAD

The ongoing digital transformation in dentistry has raised the question of whether CAD technology will eventually replace conventional prosthodontic design. Our study found that 88.7% of students believe that digital design could replace conventional methods in the future, a claim also sustained by multiple studies showing the superiority of CAD in terms of accuracy, efficiency, and predictability. Other authors analyzed digital versus conventional implant prosthetic workflows and concluded that digital workflows significantly reduce treatment time and costs while maintaining high accuracy and predictability [3]. Similarly, students in our study who supported CAD replacement associated it with greater accuracy (*p* = 0.020), predictability (*p* = 0.048), and sustainability (*p* = 0.032). Additionally, different studies emphasized the evolution of intraoral scanners, demonstrating how digital impressions have revolutionized prosthodontic workflows and patient experiences [5]. This shift has contributed to the perception among students that digital workflows are more efficient and may eventually replace conventional techniques. Studies further emphasized the clinical benefits of digital impressions for implant-supported prostheses, noting improved marginal fit, clinical outcomes, and overall patient experience [5,13].

However, despite these advancements, certain studies highlight challenges associated with fully replacing conventional design. Authors noted that while digital technology improves efficiency, students require additional training to develop proficiency in CAD software [10]. This aligns with concerns raised in our study, where a subset of students (11.3%) expressed skepticism regarding the complete replacement of conventional design, potentially due to the learning curve and initial investment required for CAD technology.

Furthermore, investigations into the fit of zirconia prostheses fabricated using both digital and conventional impressions emphasized that while digital techniques offer superior reproducibility, traditional methods still provide reliable results [11]. Similarly, others pointed out that while CAD/CAM technology enhances precision and workflow efficiency, its implementation requires substantial financial investment in equipment and training [9].

Our study’s findings contribute to this ongoing debate, demonstrating that while students acknowledge the advantages in digital design, the complete replacement of conventional workflows may be contingent on factors such as training, cost, and institutional readiness. As digital dentistry continues to advance, dental curricula must strike a balance between integrating CAD technology and preserving foundational knowledge of conventional techniques, ensuring that future dental professionals are equipped with a comprehensive skill set.

### 4.3. Interest in Future Use of Digital Design

The increasing integration of digital technologies in dental education has led to a growing interest among students in adopting CAD technology in their future practice. Our study revealed that 96.5% of students expressed a strong interest in integrating digital design into their university education and future dental careers. This aligns with some findings that reported that a higher level of understanding of digital dentistry was associated with a greater intention to use digital technology in professional practice [6].

However, while this study [6] found a direct correlation between students’ digital knowledge and their willingness to adopt digital workflows, our study did not identify a statistically significant relationship between students’ perceived understanding of theoretical concepts (both in conventional and digital prosthodontic design) and their interest in CAD technology. Instead, we observed that students who believed digital design allowed easier pattern manipulation were significantly more likely to express interest in future integration of CAD into their education and clinical practice (*p* = 0.021). This suggests that students’ practical experiences with digital workflows may play a more influential role in shaping their future preferences rather than their theoretical knowledge alone.

Similar observations were made by others, who found that students preferred digital evaluation tools over traditional methods for preparation assessment [7]. Authors also emphasized that three-dimensional digital technologies significantly improve dental training, making them more appealing to students [8]. Additionally, another study reported that students who had hands-on experience with digital impression techniques displayed a more positive attitude toward adopting digital workflows in their future careers [16].

Despite these promising trends, some studies suggest that barriers to digital adoption still exist. It was highlighted that while digital technology improves efficiency and accuracy, the initial investment in training and equipment can be a limiting factor for widespread adoption [9]. Similarly, others noted that although students found digital workflows more efficient, some still required additional training to gain full proficiency [10]. These findings emphasize the need for comprehensive digital training programs to ensure that students gain both theoretical and practical expertise in CAD technology.

Our study demonstrates that while the vast majority of students recognize the benefits of digital design and despite the enthusiasm, barriers to digital adoption remain. Their motivation to integrate it into their future practice depends more on their direct experience with its advantages rather than their theoretical understanding. These insights highlight the importance of expanding hands-on digital training within dental curricula to further enhance students’ confidence and interest in digital workflows

### 4.4. Time Allocated by the University

The implementation of digital technologies in dental education requires sufficient training time for students to develop both theoretical understanding and practical proficiency. Our study found that students who believed they had acquired sufficient theoretical knowledge and practical skills in digital design were significantly more likely to consider the allocated training time as adequate (*p* < 0.001). However, it was reported that many students viewed the time allocated for digital training in dental curricula as insufficient, potentially undermining their confidence in applying these technologies in clinical settings [6]. Similarly, it was found that students often struggled to gain proficiency in digital prosthodontic design due to limited hands-on experience during their training [9]. These findings align with concerns raised in broader dental education literature regarding the challenge in balancing traditional and digital techniques within an already dense curriculum. The dense structure of dental curricula, requiring mastery of multiple disciplines, often limits time available for digital training [6]. Additionally, authors highlighted that overcrowded curricula, additional costs, and logistical challenges related to integrating digital workflows into preclinical and clinical training may limit the ability of universities to provide extended CAD training [10].

Our study also suggests that students’ perception of training adequacy may be influenced by their level of confidence in using digital technology. While those who felt competent in digital workflows generally regarded the allocated time as sufficient, others may have needed more guided practice to reach a similar level of confidence. This discrepancy underscores the importance of flexible and adaptive training models that ensure all students—regardless of prior experience—receive the necessary exposure to digital design.

In conclusion, while our findings indicate that most students considered the current training time sufficient, comparisons with other studies suggest that further optimization of digital education is needed: improvements can be made by expanding digital training opportunities, incorporating self-paced learning resources, and ensuring structured exposure to CAD/CAM technologies.

### 4.5. Practical Semester Exams: Student Grades and Tasks Completion Time

The evaluation of student performance in practical semester exams provides valuable insights into the effectiveness of digital versus conventional design training. In our study, the grades obtained by students in the digital design exam were significantly higher (median grading score = 8.23) compared to those obtained in the conventional exam (median grading score = 7.61), (*p* < 0.001). We also found that students who believed that they acquired sufficient understanding of theoretical concepts in CAD or practical skills in CAD had significantly higher grades in digital design (*p* = 0.028; *p* = 0.001) in comparison to the students who considered that they did not acquire enough skills. These results suggest that students may have found digital workflows more intuitive and precise, contributing to improved academic performance. These ideas align with previous studies that compared student outcomes in digital versus conventional prosthodontic design. Students generally performed better when using CAD/CAM technology due to its standardized approach, which minimizes manual errors [10]. Similarly, others highlighted that digital workflows lead to more consistent results in prosthodontic design, reinforcing our findings that students achieved higher grades when assessed using digital methods [3].

In addition to grading performance, our study also assessed the time required to complete practical tasks. Students spent significantly less time on digital projects compared to conventional projects, with an average difference of 22–41 min. This result confirms the time efficiency of digital workflows, as previously reported in the literature. For instance, studies found that students required approximately 30 min longer to complete conventional wax-ups than digital designs, further supporting the idea that CAD/CAM technology accelerates the design process [10]. Additionally, it was demonstrated that digital workflows reduce overall treatment time without compromising quality, which is consistent with our findings [3].

While digital workflows appear to provide advantages in terms of efficiency and performance, it is essential to acknowledge the initial learning curve associated with CAD technology. Studies emphasized that students often require additional training to develop full proficiency in digital design [9,11]. However, our results suggest that once students become familiar with digital tools, they can execute projects more efficiently than with traditional methods. Furthermore, our findings indicate that students who perceived themselves as having acquired sufficient theoretical and practical skills in digital design were more likely to complete their tasks faster and receive higher grades. This supports the idea that confidence in digital workflows plays a crucial role in performance outcomes. Similarly, it was found that students who engaged more extensively with digital tools displayed greater proficiency and efficiency in their work [8].

Students’ enthusiasm and performance indicate a strong basis for expanding digital training in dental education. Other studies similarly report that exposure to digital tools enhances motivation and fosters long-term interest in dentistry [17]. Literature also highlights that digital technologies improve spatial cognition and psychomotor skills—essential for prosthodontics [6,18,19,20,21,22,23,24]. Structured digital education has been linked to increased preparedness for clinical tasks compared to conventional-only training [25]. Additionally, digital workflows have been shown to improve clinical accuracy, reduce procedural time, and increase patient satisfaction [26,27,28].

The observed students’ preference for digital methods reflects a generational shift in learning, as today’s digital-native learners adapt quickly to virtual tools and value visual feedback and interactivity—features inherent to digital systems [4,29,30,31]. These tendencies should be met with flexible, future-oriented curricula. Notably, participants in this study achieved higher grades in digital design exams compared to conventional ones. However, digital integration faces challenges, including equipment costs, faculty training demands, and the need to maintain manual competencies. Balancing innovation with core clinical skills remains essential. To support effective learning, digital content should be progressively integrated across the curriculum, from preclinical training to clinical application. Tools like adaptive learning systems, AR simulations, and digital assessments can enhance engagement. In this line, our results showed that students completed digital designs more efficiently than conventional ones during practical exams.

It can be asserted that our study indicates that digital prosthodontic design offers significant advantages over conventional methods in terms of student performance and time efficiency. The higher grades and reduced task completion times observed in digital design assessments indicate a positive shift toward CAD-based workflows in dental education. However, the integration of digital training should be carefully structured to ensure that all students develop the necessary skills to fully benefit from these technological advancements.

### 4.6. Limits of the Study

The relatively small, homogenous sample—limited to second-year students from a single institution—may limit the generalizability of results. Broader, multi-institutional studies are needed to improve external validity. Moreover, the reliance on self-reported survey data introduces potential bias, as responses may be influenced by individual experiences or subjective interpretations. Future studies incorporating objective assessments or follow-up evaluations could offer a clearer view of student competency and long-term digital adoption. Additionally, practical semester exams conducted in a controlled university laboratory setting may not fully represent actual clinical practice, where factors such as patient variability, workflow constraints, and material limitations play a significant role. Long-term studies assessing skill retention in clinical settings would be beneficial. The rapid advancement in digital dentistry presents another challenge, as curricula may struggle to stay aligned with technological developments. Continuous updates and faculty development are essential to keep education aligned with industry advancements. Finally, although a time advantage was observed for digital workflows, it may reflect differing levels of prior exposure to digital tools among students. Further research should examine whether additional training could equalize performance across varying experience levels.

Despite these limitations, this study contributes to the discourse on digital education in prosthodontics, highlighting areas for refinement and supporting the advancement in digitally integrated curricula.

### 4.7. Future Perspectives

The integration of digital technologies into university dental education continues to advance, offering new opportunities for improving both teaching and clinical training. Our findings indicate a strong student preference for digital workflows, emphasizing the need to further develop curricula, faculty expertise, and technological access to support this transition. Expanding structured digital training within dental curricula is essential. Although students reported adequate skills in digital design, enhanced educational strategies—such as hands-on sessions, virtual simulations, and interactive modules—can strengthen practical competence. Emerging tools like augmented reality (AR) and artificial intelligence (AI) may further improve clinical preparedness [5]. On the other hand, equitable access to digital technologies remains a challenge, as CAD/CAM systems and scanners involve significant costs for many universities [9]. Institutional investment and partnerships with industry could be crucial in delivering affordable, scalable digital training.

From a research standpoint, future studies should examine the long-term effects of digital education on clinical performance and practice integration. Our future studies could include students in later academic years to enable a more comprehensive comparison of perspectives, potentially revealing key differences across educational stages and offering deeper insights into students’ evolving attitudes and needs. Comparative studies assessing graduates trained with digital versus conventional methods could offer meaningful insights into real-world outcomes. Additionally, with technology evolving rapidly, continuous faculty development is crucial. Ongoing training in CAD/CAM and related innovations will ensure that educators remain current and capable in delivering high-quality instruction [6].

## 5. Conclusions

Considering the study’s results and limitations, the following conclusions were drawn:

- The participating students responded positively regarding their knowledge and practical skills in both conventional and digital prosthodontic design;
- A favorable attitude toward digital design was observed, to the detriment of conventional design;
- Students who expressed greater interest in the future of CAD more frequently emphasized its advantages over conventional design, including improved workflow, enhanced accuracy, sustainability, and ease of pattern manipulation;
- Grades achieved in the digital design practical exam were significantly higher than those in the conventional design exam.

These findings may reflect a great openness and availability among participating students for digital technologies.

## Figures and Tables

**Table 1:** Distribution of the students according to the answers regarding the advantages of CAD versus conventional design and future replacement of conventional design with CAD.

Conventional Design Replacement/Digital Design Advantages Workflow Improvement	Replace—Disagree		Replace—Agree		*p* *
	Nr.	%	Nr.	%	
Absent	1	6.3%	2	1.6%	0.305
Present	15	93.7%	123	98.4%	
Conventional design replacement/digital design advantages Easier manipulation of patterns	Replace—Disagree		Replace—Agree		p *
	Nr.	%	Nr.	%	
Absent	1	6.3%	38	30.4%	0.070
Present	15	93.7%	87	69.6%	
Conventional design replacement/digital design advantages Better accuracy	Replace—Disagree		Replace—Agree		*p* *
	Nr.	%	Nr.	%	
Absent	3	18.8%	3	2.4%	**0.020**
Present	13	81.2%	122	97.6%	
Conventional design replacement/digital design advantages Predictable success	Replace—Disagree		Replace—Agree		*p* *
	Nr.	%	Nr.	%	
Absent	3	18.8%	5	4%	**0.048**
Present	13	81.2%	120	96%	
Conventional design replacement/digital design advantages Time efficiency	Replace—Disagree		Replace—Agree		*p* *
	Nr.	%	Nr.	%	
Absent	2	12.5%	8	6.4%	0.317
Present	14	87.5%	117	93.6%	
Conventional design replacement/digital design advantages Better communication	Replace—Disagree		Replace—Agree		p *
	Nr.	%	Nr.	%	
Absent	1	6.3%	10	8%	1.000
Present	15	93.7%	115	92%	
Conventional design replacement/digital design advantages Sustainability	Replace—Disagree		Replace—Agree		*p* *
	Nr.	%	Nr.	%	
Absent	4	25%	8	6.4%	**0.032**
Present	12	75%	117	93.6%	

* Fisher’s exact test.

**Table 2:** Distribution of the students according to the answers regarding the advantage of easier pattern manipulation as a benefit of digital design and interest in future integration of CAD technologies into university education and dental practice.

Interest in Future Use of Digital Design/Digital Design Advantages Easier Manipulation of Patterns	Interest— Disagree		Interest— Agree		*p* *
	Nr.	%	Nr.	%	
Absent	4	80%	35	25.7%	**0.021**
Present	1	20%	101	74.3%	

* Fisher’s exact test.

**Table 3:** Distribution of the students according to their answers regarding the acquirement of theoretical knowledge and practical skills and time allocated for these aspects by the university.

Sufficient Understanding of Theoretical Concepts in Conventional Design/ Sufficient Time Allocated by the University for Conventional Design	Time— Disagree		Time— Agree		*p* *
	Nr.	%	Nr.	%	
Skills—Disagree	1	10%	9	6.9%	0.533
Skills—Agree	9	90%	122	93.1%	
Sufficient acquired practical skills in conventional design/ Sufficient time allocated by the university for conventional design	Time—Disagree		Time— Agree		*p* *
	Nr.	%	Nr.	%	
Skills—Disagree	1	10%	29	22.1%	0.689
Skills—Agree	9	90%	102	77.9%	
Sufficient understanding of theoretical concepts in digital design/ Sufficient time allocated by the university for digital design	Time—Disagree		Time— Agree		*p* *
	Nr.	%	Nr.	%	
Skills—Disagree	8	33.3%	4	3.4%	**<0.001**
Skills—Agree	16	66.7%	113	96.6%	
Sufficient practical skills in digital design/ Sufficient time allocated by the university for digital design	Time—Disagree		Time— Agree		*p* *
	Nr.	%	Nr.	%	
Skills—Disagree	14	58.3%	17	14.5%	**<0.001**
Skills—Agree	10	41.7%	100	85.5%	

* Fisher’s exact test.

**Table 4:** Comparison of the grading scores in the analyzed students.

Parameter	Mean ± SD	Median (IQR)	Min	Max	*p* *
Grade—Conventional	7.68 ± 1.91	8 (6–9)	4	10	**<0.001**
Grade—CAD	8.3 ± 1	8 (7.75–9)	6	10	
Difference	0.61 ± 1.65	0 (−1–2)	−3	6	-

* Related-samples Wilcoxon signed rank test.

**Table 5:** Comparison of the grading scores in the analyzed students according to their opinion about skill acquirement in conventional and digital design.

Sufficient Understanding of Theoretical Concepts in Conventional Design/Grade	Mean ± SD	Median (IQR)	Mean Rank	*p* *
Disagree	7.6 ± 2.011	8 (5.75–9.25)	69.15	0.880
Agree	7.69 ± 1.91	8 (6–9)	71.14	
Sufficient acquired practical skills in conventional design/Grade	Mean ± SD	Median (IQR)	Mean rank	*p* *
Disagree	7.43 ± 1.97	7.5 (5.75–9)	65.60	0.408
Agree	7.75 ± 1.9	8 (6–9)	72.40	
Sufficient understanding of theoretical concepts in CAD/Grade	Mean ± SD	Median (IQR)	Mean rank	*p* *
Disagree	7.66 ± 1.15	7 (7–8.75)	47.12	**0.028**
Agree	8.36 ± 0.97	8 (8–9)	73.22	
Sufficient acquired practical skills in CAD/Grade	Mean ± SD	Median (IQR)	Mean rank	*p* *
Disagree	7.77 ± 1	7 (7–9)	50.90	**0.001**
Agree	8.45 ± 0.96	8 (8–9)	76.66	

* Mann–Whitney U test.

**Table 6:** Time scores registered for the conventional and digital design during the practical exam (in minutes).

Prosthodontic Design	Mean	SD	Min	Max
Conventional projects	74	6	36	110
Digital projects	42	10	14	69

## Data Availability

The original contributions presented in the study are included in the article and Supplementary Materials; further inquiries can be directed to the corresponding author/s.

## Supplementary Materials

The following supporting information can be downloaded at: https://www.mdpi.com/article/10.3390/dj13060242/s1, Table S1: The questionnaire used to evaluate the opinion of dental students on the use of digital design vs conventional design in prosthodontics; the questionnaire was administrated as a Google forms and sent via email.

## References

[1] Ahlholm P., Sipilä K., Vallittu P., Jakonen M., Kotiranta U. (2018). Digital Versus Conventional Impressions in Fixed Prosthodontics: A Review. J. Prosthodont..

[2] Burhardt L., Livas C., Kerdijk W., van der Meer W.J., Ren Y. (2016). Customized CAD-CAM healing abutment for delayed loaded implants. J. Prosthet. Dent..

[3] Joda T., Brägger U. (2016). Digital vs. conventional implant prosthetic workflows: A cost/time analysis. Clin. Oral Implant. Res..

[4] Gjelvold B., Chrcanovic B.R., Korduner E.K., Collin-Bagewitz I., Kisch J. (2016). Intra-oral digital impression technique compared to conventional impression technique. A randomized clinical trial. J. Prosthodont..

[5] Mangano F., Gandolfi A., Luongo G., Logozzo S. (2017). Intraoral scanners in dentistry: A review of the current literature. BMC Oral Health.

[6] Sheba M., Comnick C., Elkerdani T., Ashida S., Zeng E., Marchini L. (2021). Students’ perceptions and attitudes about digital dental technology is associated with their intention to use it. J. Dent. Educ..

[7] Hamil L.M., Mennito A.S., Renne W.G., Vuthiganon J. (2014). Dental students’ opinions of preparation assessment with E4D compare software versus traditional methods. J. Dent. Educ..

[8] Patel N. (2010). Integrating three-dimensional digital technologies for comprehensive implant dentistry. J. Am. Dent. Assoc..

[9] Cooper L.F., Ludlow M.E. (2017). The Current Impact of Digital Technology in Prosthodontics. American College of Prosthodontists. https://www.prosthodontics.org/assets/1/7/Digital_White_Paper_r1.pdf.

[10] Douglas R.D., Hopp C.D., Augustin M.A. (2014). Dental students’ preferences and performance in crown design: Conventional wax-added versus CAD. J. Dent. Educ..

[11] Almeida e Silva J.S., Erdelt K., Edelhoff D., Araújo É., Stimmelmayr M., Vieira L.C.C., Güth J.F. (2014). Marginal and internal fit of four-unit zirconia fixed dental prostheses based on digital and conventional impression techniques. Clin. Oral Investig..

[12] Fasbinder D.J. (2012). Digital dentistry: Innovation for restorative treatment. Compend. Contin. Educ. Dent..

[13] Corsalini M., Barile G., Ranieri F., Morea E., Corsalini T., Capodiferro S., Palumbo R.R. (2024). Comparison between Conventional and Digital Workflow in Implant Prosthetic Rehabilitation: A Randomized Controlled Trial. J. Funct. Biomater..

[14] Moraru A. (2020). Research on the Fabrication of Dental Prostheses Using Selective Laser Melting and Other Additive Technologies. Ph.D. Thesis.

[15] Alghazzawi T.F. (2016). Advancements in CAD/CAM technology: Options for practical implementation. J. Prosthodont. Res..

[16] Zitzmann N.U., Kovaltschuk I., Lenherr P., Dedem P., Joda T. (2017). Dental students’ perceptions of digital and conventional impression techniques: A randomized controlled trial. J. Dent. Educ..

[17] Goncharuk-Khomyn M., Tsiuman M., Solonenko A. (2024). Digital dental technologies as a factor in the educational motivation of students in a disrupted learning environment. J. Int. Dent. Med. Res..

[18] Hall M.A., Mahmoud A.Z., Mohamed O.S., Karawia I. (2024). Knowledge, Awareness, and Perception of Dental Students Regarding Digital Dentistry in Egypt: A Cross-Sectional Study. Cureus.

[19] Yaparathna N., Udayamalee I., Gray M., Cameron A., Evans J., Abuzar M. (2025). Technology-Enabled Active Learning: Assessment of Dentistry Students’ Perception of Digital Prosthodontic Workflow. Dent. J..

[20] Kakti A., Alhisan A.M., Alammar A.M., Almakadi F.S., Alibrahim K.E., Alkraidees M., Alshahrani O.D. (2022). Knowledge and Perception of Senior Dental Students Regarding Digital Dentistry and Its Use in Prosthodontics. Ann. Dent. Spec..

[21] Gad M.M., Al Shehab S.S., Alshaikhnasser F.Y., Alboryh S.Y., Alkhalaf A.I., Khan S.Q., Alakloby B.O., Alharbi H.M., Alhorish N., Alrajhi S. (2025). Dental Students’ Awareness Regarding the Implementation of Digital Dentistry in Prosthodontics—A Questionnaire-Based Study. Prosthesis.

[22] Schlenz M.A., Michel K., Wegner K., Schmidt A., Rehmann P., Wöstmann B. (2020). Undergraduate dental students’ perspective on the implementation of digital dentistry in the preclinical curriculum: A questionnaire survey. BMC Oral Health.

[23] Revilla-León M., Özcan M. (2019). Additive Manufacturing Technologies Used for Processing Polymers: Current Status and Potential Application in Prosthetic Dentistry. J. Prosthodont. Off. J. Am. Coll. Prosthodont..

[24] Dawood A., Marti Marti B., Sauret-Jackson V., Darwood A. (2015). 3D printing in dentistry. Br. Dent. J..

[25] Mahato M., Hota S., Jain A., Dutta D., Bhushan P., Raut A. (2024). Comparison of Conventional and Digital Workflows in the Fabrication of Fixed Prostheses: A Systematic Review. Cureus.

[26] Wang X., Wan Z., Feng X., Zhu Z. (2024). Perceptions of a Digital Dental Technology Curriculum: A Qualitative Study of Dental Technology Students. Eur. J. Dent. Educ. Off. J. Assoc. Dent. Educ. Eur..

[27] Cristache C., Totu E., Mihuţ T., Iorgulescu G., Pintilie I., Burlacu-Vătămanu O.-E., Burlibasa M. (2020). Digital Versus Analog în Medicina Dentară—Partea I. XV. 41–44.—Part I. ResearchGate. https://www.researchgate.net/publication/376237800.

[28] Cristache C., Burlibașa M., Ionescu C. (2017). The use of CAD-CAM technology in removable prosthodontics. Int. J. Prosthodont..

[29] Giménez B., Özcan M., Martínez-Rus F., Pradíes G., Salinas T.J. (2015). Accuracy of a digital impression system based on active wavefront sampling technology for implants considering operator experience, implant angulation, and depth. Clin. Implant. Dent. Relat. Res..

[30] Lee S.J., MacArthur R.X., Gallucci G.O. (2013). An evaluation of student and clinician perception of digital and conventional implant impressions. J. Prosthet. Dent..

[31] Shinde P., Sarda A., Memapally H., Srivastava H., Aysha R., Nile S. (2024). Which one is better? Digital v/s conventional impression for fixed prosthodontics: A review. Int. J. Appl. Dent. Sci..

