# Supplementary material for: Evaluation of Undergraduate Dental Students’ Opinions on the Use of Digital Versus Conventional Design in Prosthodontics
Source: Dent J (Basel). 2025 May 29;13(6):242. doi: 10.3390/dj13060242 (PMC12192495; doi:10.3390/dj13060242)
Supplement: Supplementary file 1 [file dentistry-13-00242-s001.zip › dentistry-3613842-supplementary.pdf]

Supplementary File:

Table S1. The questionnaire used to evaluate the opinion of dental students on the use of digital design vs conventional design in prosthodontics; the questionnaire was administrated as a Google forms and sent via email

Investigated aspects	Questions (Q) and possible answers
**(1) Socio-demographic details**	**Q1**. Please enter your age
	**Q2.** Please enter your gender
	a. female
	b. male
	c. other / I prefer not to answer
	**Q3**. Please enter your study year
**(2) Assessment of participants' opinion on their knowledge and practical skills regarding conventional and digital design in prosthetic dentistry**	**Q4.** I consider that I have **acquired sufficient understanding** following the completion of university theoretical courses in the conventional fabrication of wax patterns for dental prosthetic restorations:
	a. I agree
	b. I disagree
	**Q5.** I consider that I have **acquired sufficient practical skills** for the conventional workflow in the fabrication of wax patterns for dental prosthetic restorations during the practical training activities at the university:
	a. I agree
	b. I disagree
	**Q6.** I consider that I have **acquired sufficient understanding** following the completion of theoretical courses in the digital design concept (CAD/Computer-Aided Design) for projecting dental prosthetic restorations:
	a. I agree
	b. I disagree
	**Q7.** I consider that I have **acquired sufficient practical skills** for the digital design (CAD) of dental prosthetic restorations during the practical training activities at the university:
	a. I agree
	b. I disagree
	**Q8.** I consider that the amount of time allocated by the university for **theoretical courses and practical training** in **digital design** within the field of prosthodontics was sufficient for me:
	a. I agree
	b. I disagree
	**Q9.** I consider that the amount of time allocated by the university for **theoretical courses and practical training in in the conventional design** within the field of prosthodontics was sufficient for me:
	a. I agree
	a. I disagree
**(3) Assessment of participants' opinion on certain particularities of CAD and conventional design in prosthetic dentistry**	**Q10.** I consider that CAD/CAM technology is **useful** for medical applications:
	a. I agree
	b. I disagree
	**Q11.** I consider that CAD technology could improve the **workflow stages** involved in obtaining dental prosthetic restorations:
	a. I agree
	b. I disagree
	**Q12.** I consider that **manipulating** patterns of dental prosthetic restorations in the conventional workflow can be done more easily compared to the one performed on the computer (CAD):

	a. I agree
	b. I disagree
	**Q13.** I consider that the use of CAD technology could limit errors in obtaining dental prosthetic restorations, increasing their **accuracy:**
	a. I agree
	b. I disagree
	**Q14:** I consider that the use of CAD technology could increase my **level of confidence** regarding the **success** of prosthetic treatment:
	a. I agree
	b. I disagree
	**Q15.** I consider that creating/designing wax patterns for dental prosthetic restorations in the conventional workflow is **more time-consuming** compared to that done on the computer (CAD):
	a. I agree
	b. I disagree
	**Q16.** I consider that CAD technology can facilitate **communication** between the dentist and dental technician, aiming to achieve dental prosthetic restorations with increased strength and precision:
	a. I agree
	b. I disagree
	**Q17.** I consider that the use of CAD technology could enhance and optimize the utilization of **resources** (financial, material, human) associated with obtaining dental prosthetic restorations:
	a. I agree
	b. I disagree
**(4) Interest in the future use of digital technology**	**Q18.** I believe **that digital design could replace conventional design** for dental prosthetic restorations in the future:
	a. I agree
	b. I disagree
	**Q19.** I am interested in integrating CAD technology into my university education and future dental practice:
	a. I agree
	b. I disagree
